# Gamma radiation sterilization of N95 respirators leads to decreased respirator performance

**DOI:** 10.1371/journal.pone.0248859

**Published:** 2021-04-08

**Authors:** Haedi E. DeAngelis, Anne M. Grillet, Martin B. Nemer, Maryla A. Wasiolek, Don J. Hanson, Michael A. Omana, Andres L. Sanchez, David W. Vehar, Paul M. Thelen

**Affiliations:** Sandia National Laboratories, Albuquerque, NM, United States of America; VIT University, INDIA

## Abstract

In response to personal protective equipment (PPE) shortages in the United States due to the Coronavirus Disease 2019, two models of N95 respirators were evaluated for reuse after gamma radiation sterilization. Gamma sterilization is attractive for PPE reuse because it can sterilize large quantities of material through hermetically sealed packaging, providing safety and logistic benefits. The Gamma Irradiation Facility at Sandia National Laboratories was used to irradiate N95 filtering facepiece respirators to a sterilization dose of 25 kGy(tissue). Aerosol particle filtration performance testing and electrostatic field measurements were used to determine the efficacy of the respirators after irradiation. Both respirator models exhibited statistically significant decreases in particle filtering efficiencies and electrostatic potential after irradiation. The largest decrease in capture efficiency was 40–50% and peaked near the 200 nm particle size. The key contribution of this effort is correlating the electrostatic potential change of individual filtration layer of the respirator with the decrease filtration efficiency after irradiation. This observation occurred in both variations of N95 respirator that we tested. Electrostatic potential measurement of the filtration layer is a key indicator for predicting filtration efficiency loss.

## Introduction

The Coronavirus Disease 2019 (COVID-19) pandemic has strained healthcare systems and has led to a shortage of personal protective equipment (PPE). PPE is crucial to protect healthcare workers and maintain the highest quality patient care. Various non-traditional solutions have been proposed to relieve shortages, such as PPE sterilization and reuse, fabric face masks, and 3D-printed equipment [1–3]. Health care systems will likely employ various methods to address the PPE shortage based on their resources and capabilities. Some sterilization methods have shown promising results; however, multiple sterilization methods have been shown to degrade the filtration ability of respirators and need to be assessed for performance to ensure proper protection for wearers [4–8]. There are several other factors also need to be considered, including, PPE handling requirements, throughput, the ability to fully penetrate and decontaminate, shadowing, and safety [9–11]. Comparisons of various sterilization methods have been compiled for various performance factors, such as filtration efficiency, respirator integrity, and handling requirements [12–14]. Gamma sterilization can be found in one such compilation [12].

Gamma radiation sterilization is a commonly used method for sterilization of medical equipment [15] and large scale facilities exists across the United States [16]. Gamma sterilization of used PPE has been proposed as one method to enhance the supply of PPE [17]. Gamma sterilization has been shown to inactivate many pathogens, including the Severe Acute Respiratory Syndrome Coronavirus 1 (SARS-CoV-1) at a dose of 10 kGy(tissue) [18]. At the time of writing this paper, our literature review has not shown the inactivation dose of SARS-CoV-2, the virus that causes COVID-19. Gamma sterilization has several key benefits for sterilizing N95 respirators for reuse: 1) pre-existing, large scale/volume facilities, 2) the ability to sterilize densely packed PPE and 3) the penetration ability to sterilize objects while they reside inside hermetically sealed containers. This minimizes exposure to personnel during transportation and sterilization of contaminated material. These key benefits unique to gamma radiation sterilization justify closer examination.

It is important to note that gamma sterilization should be approached with caution due to the unique effects of ionizing radiation on materials. Gamma radiation is known to modify the structure and properties of polymers and can adversely affect the filter materials in several ways, such as enhanced polymer oxidation, polymer chain scission or cross-linking which can degrade mechanical performance of the polymer [19–21]. Because of this, some N95 manufacturers recommend against gamma sterilization [22]. However, a potentially more impactful effect of gamma radiation on N95 respirators is the removal of charge in the electret layer. Many N95 respirators rely on electric charge for particle or pathogen removal [23]. Most N95 respirators use a meltblown, electret material that employs electrostatic forces to increase particle capture. Some respirators use a non-woven polymer fiber fabric as a filter.

Unlike other sterilization methods, there are only a small number of studies that have examined gamma sterilization for N95 respirators. Due to the recent importance of understanding respirator sterilization and reuse, most of these studies were performed simultaneously to each other and to this work. One observation common to these studies was a reduction in filtration efficiency after gamma sterilization, especially for particle sizes between 100 to 300 nm [17, 23, 24]. This range is especially important because it overlaps with the aerosol particle sizes produced by coughing [25]. Of note, researchers found no significant physical or chemical changes in respirators after gamma irradiation [23, 24]. One study that is especially complementary to this work linked the reduction in filtration efficiency with a change in electret non-contact electrostatic potential by taking measurements after exposure inside of a Training, Research, Isotopes, General Atomics (TRIGA) reactor. The electrostatic potential measurement was done on complete filters consisting of four layers. It was also demonstrated that recharging the electret layer leads to substantial recovery of the filtration performance [26].

The key contribution of this study is an assessment of the electret and filtration properties of the two available models of respirators before and after exposure to gamma radiation in a cobalt-60 facility. Our radiation source selection was driven by the existence of numerous, large-scale, cobalt-60 sterilization facilities [16]. Note that our assessment does not determine the required dose for sterilization, but rather what effects a standard radiation sterilization dose would have on the efficacy of N95 respirators. The effects that we will assess are 1) filtration efficiency reduction as measured by aerosol penetration testing, and 2) electrostatic potential change measured by both contact and non-contact probes on each individual layer of the respirators. We successfully observed and quantified these effects and the results impact the decision to use gamma sterilization for reuse of N95 respirators.

## Materials and methods

### Respirators

**Fig 1:**
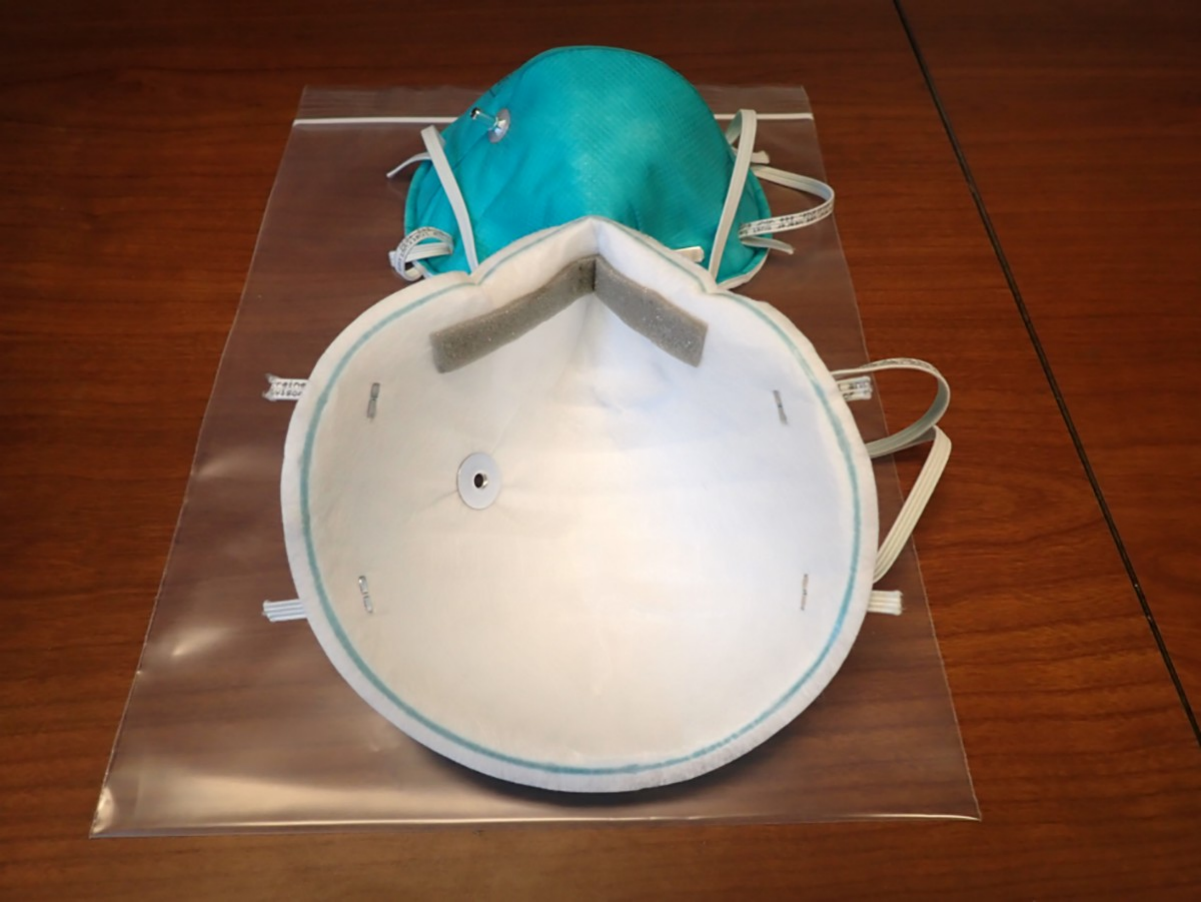
Model 1-N95 cup-shaped respirator.

**Fig 2:**
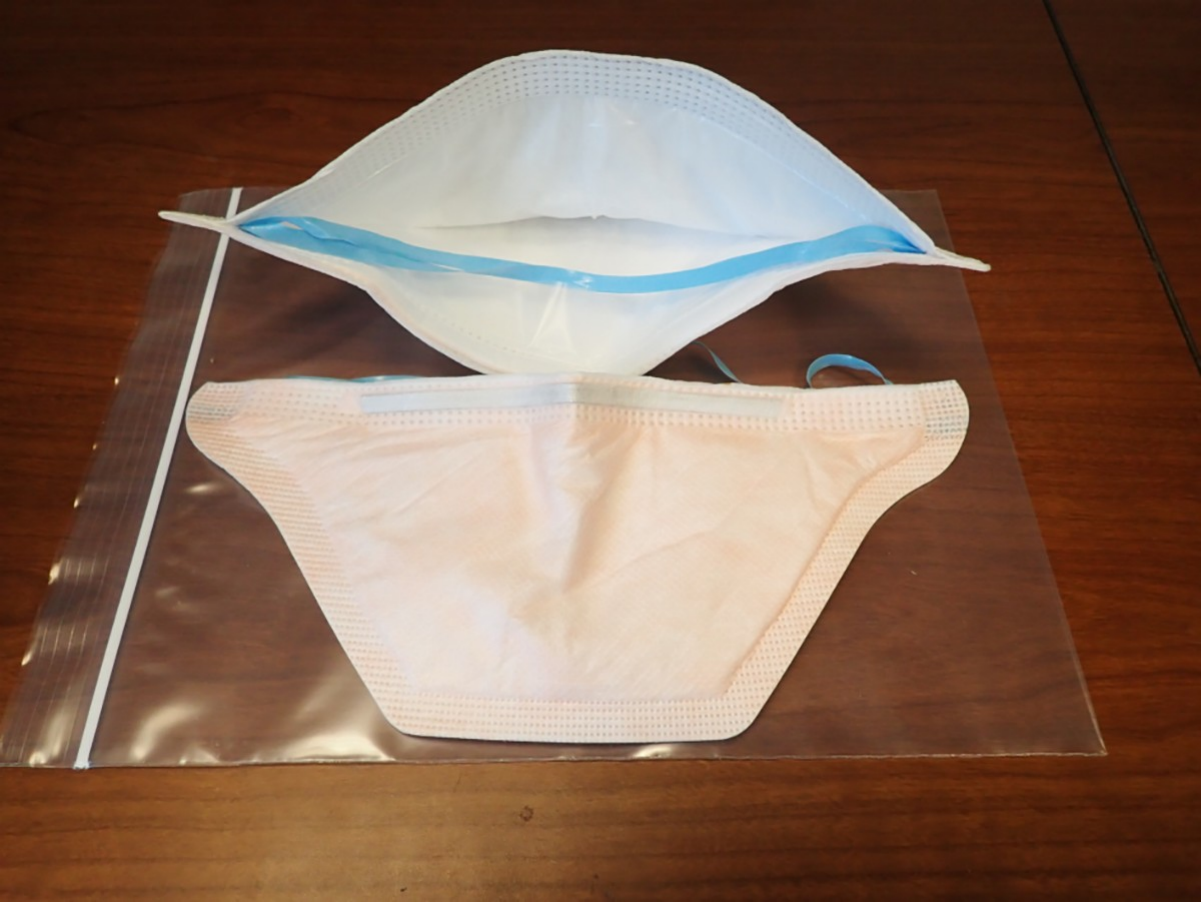
Model 2-N95 pouch/duck-bill respirator.

**Fig 3:**
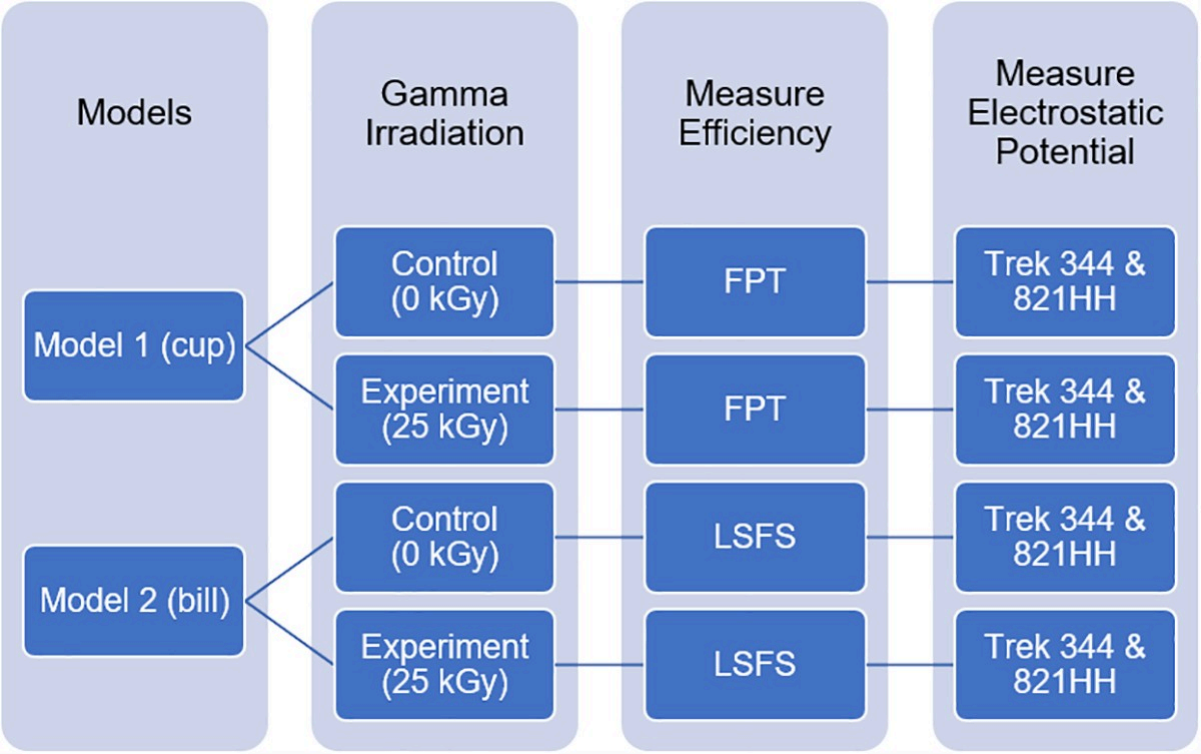
Summary of experimental methods. Abbreviations: FPT, Filter Penetration Testbed; LSFS, Large-scale filtration system.

N95 respirators meet the National Institute for Occupational Safety and Health (NIOSH) classification for air filtration; that is the respirator filters at least 95% of airborne particles of 0.3 μm in size. Many modern N95 respirator models use an electret material that uses electrostatic forces as a key mechanism to filter. The two respirators used in this study represent two different styles of N95 respirators which we will label “Model 1” and “Model 2” (the authors are willing to share specific information about the respirators to investigators who are conducting similar experiments or who are reproducing our results). Model 1 is a cup-shaped respirator (**Fig 1**). Model 2 is a pouch or duck-bill style respirator (**Fig 2)**. A total of eight new, unused respirators were used for this effort: four of each model with two control and two experimental for each model. **Fig 3**summarizes experimental methods and details of each step are given below.

### Radiation source

The N95 respirators were irradiated at the Sandia National Laboratories Gamma Irradiation Facility (GIF) [27]. This facility uses the cobalt-60 isotope to produce 1.17 and 1.33 MeV photons. The dose rate provided by this facility can be as high as 40 Gy(CaF_2_:Mn)/s; however, for this effort we used a dose rate of 0.5 Gy(CaF_2_:Mn)/s to minimize material heating. 25 kGy(tissue) was selected as a dose for this effort because ISO11137-2:2013 specifies methods to substantiate 25 kGy(tissue) as a dose for sterility of medical devices [15]. Though lower sterilization doses can inactivate viruses and can be substantiated, many commercial irradiators are set up to target a dose of 25 kGy(tissue).

For this experiment, we chose to use the GIF’s 30-pin planar array of cobalt-60 to maximize uniformity of the dose. This geometry is most representative of commercial panoramic irradiators.

### Dosimetry

Two sets of respirators were irradiated targeting 25 kGy(CaF_2_:Mn). An ionization chamber was used to measure the dose rate around the respirators. This dose rate was used to determine the required duration to target 25 kGy(CaF_2_:Mn) (first column of **Table 1**).

**Table 1:** Alanine dosimetry measurements.

Pellet ID	Target Dose (kGy (CaF_2_:Mn))	Spectral Titration	Measured Dose (kGy (Tissue))	Estimated Uncertainty of Measured Dose
1	25	281.46	28.5	4.2%
2	25	279.41	28.2	4.2%
3	25	278.33	28.1	4.2%
4	25	274.27	27.6	4.2%
5	25	271.99	27.3	4.2%
6	25	270.26	27.0	4.2%

The actual dose applied to the respirators was later confirmed using alanine pellets as dosimeters (fifth column of **Table 1**). Three alanine dosimeters, nominally 65 mg, were irradiated in equilibrated vials with each set of respirators for a total of six dosimeters. These alanine dosimeters are referenced to National Institute of Standards and Technology (NIST) traceable ion chamber measurements. Evaluation of absorbed dose was done by Electron Paramagnetic Resonance (EPR) spectroscopy using a Bruker ELEXSYS E500 spectrometer. Measured signal as compared to a reference pellet by spectral titration to normalize for environmental conditions in the resonance cavity at the time of measurement. This technique yields a measurement precision of approximately 1% and overall measurement uncertainties within 5% (1σ). Spectral titration, in **Table 1**, is an EPR measurements compared to a reference, in this case another alanine pellet. The alanine dosimetry shows that all samples were exposed to tissue doses slightly higher than the target calcium fluoride dose to ensure we do not underestimate the degradation caused by gamma radiation.

### Aerosol filtration performance testing

Due to the difference in form factor of the two models of N95, two different existing filtration systems were used to test the filtration performance of the respirators.

**Fig 4:**
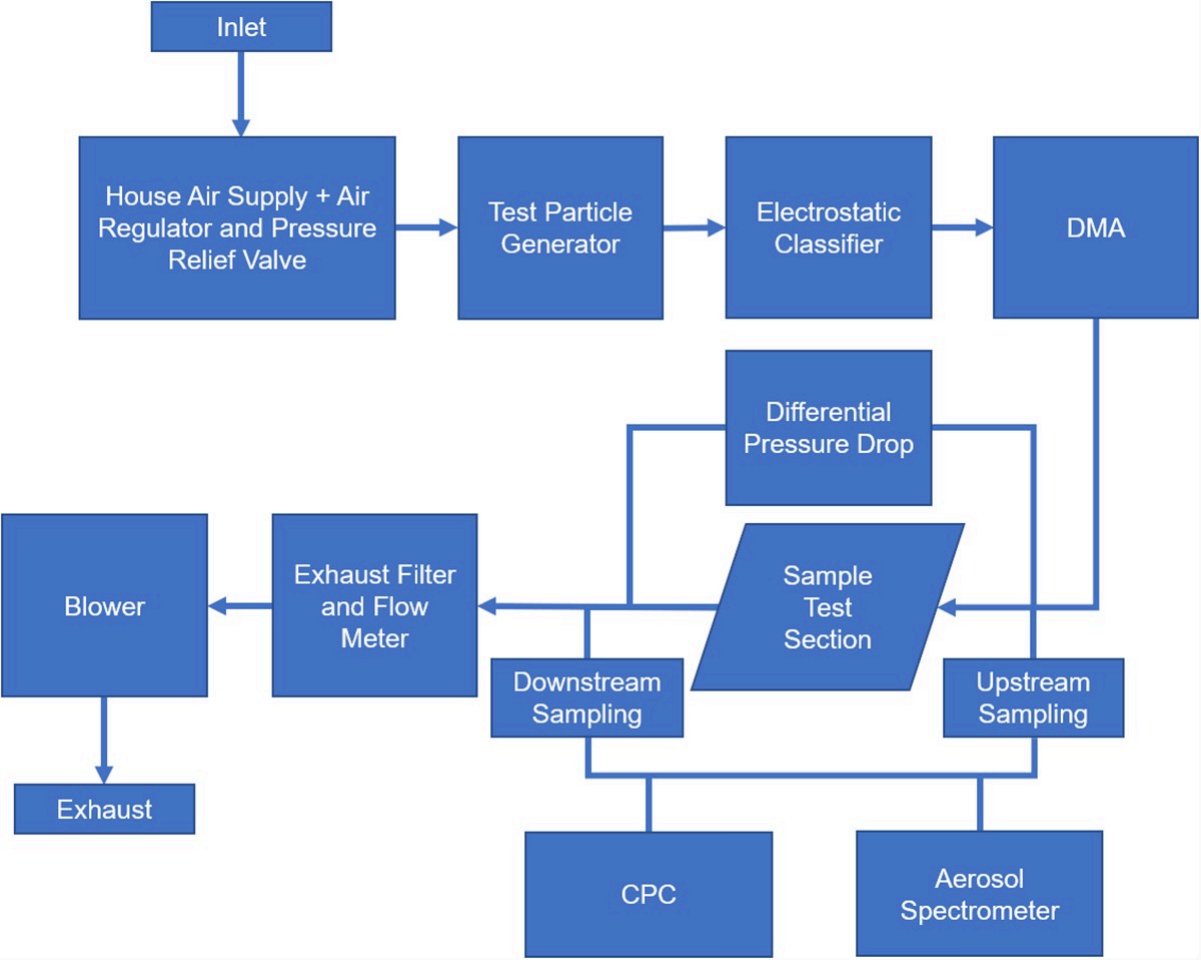
Schematic of Filter Penetration Testbed (FPT). Model 1 cup shape respirator was tested on the FPT. Abbreviations: DMA, differential mobility analyzer; CPC, condensation particle counter.

The first system is a Filter Penetration Testbed (FPT) that tests circular samples of material (**Fig 4**). The testbed was designed to follow, where possible, NIOSH guidance and the 42 Code of Federal Regulation (CFR) Part 84 [28]. The FPT performs filtration efficiency measurements on a monodisperse NaCl aerosol (generated with a TSI 3076 Atomizer) by utilizing a TSI 8020 Electrostatic Classifier and TSI 3081L Differential Mobility Analyzer (DMA) upstream from a 47 mm stainless filter housing. NIOSH guidance mentions an aerosol particle size distribution with count median diameter of 75 ± 20 nm and geometric standard deviation not exceeding 1.86 nm neutralized to the Boltzmann equilibrium state, which the FPT system measured. However, it is worth noting that the monodisperse aerosol does not capture the Food and Drug Administration (FDA) recommended mass median particle diameter of 300 nm. Additionally, the filter housing diameter used by the FPT is smaller than the mounts used by TSI 8130 (120mm). Therefore, flow rates were adjusted to achieve a filter face velocity of 17.3 cm/s, so the effect of the filter house diameter was mitigated. Finally, in order to calculate filter efficiency, concentration was measured upstream and downstream of the filter material tested by a TSI 8022 Condensation Particle Counter (CPC) and a Promo 2000 and PALAS 2300 aerosol sensor. Circular samples with a 47 mm diameter of Model 1 were tested on this machine.

**Fig 5:**
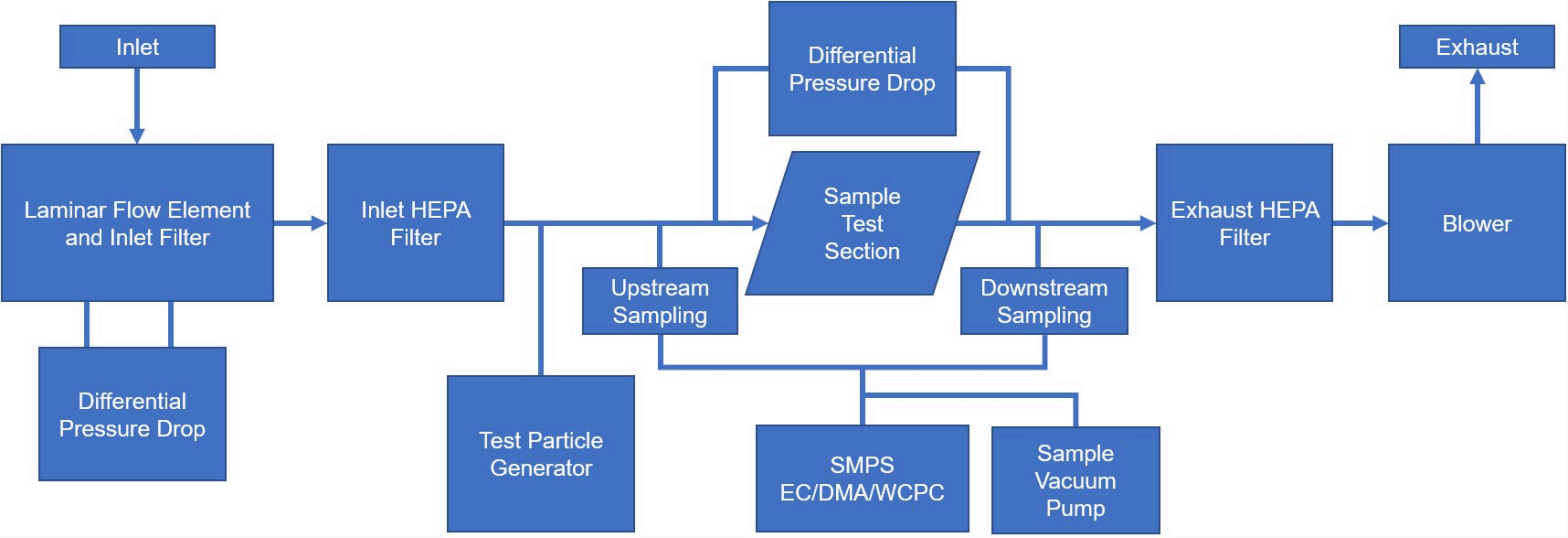
Schematic of Large-Scale Filtration System (LSFS). Model 2 bill shaped respirator was tested on the LSFS. Abbreviations: EC, electrostatic classifier; DMA, differential mobility analyzer; WCPC, water based condensation particle counter; HEPA, high-efficiency particulate air.

The second system is a large-scale filtration system (LSFS) designed to test commercial filter boxes and was modified to test bill shaped respirators (**Fig 5**). It has controlled laminar air flow, pressure drop measurements, and uses a polydisperse sodium chloride (NaCl) test aerosol. Air is filtered and enters through the Z50MH10-125F and Z50MC2-2F laminar flow elements where pressure is measured by the Omega PX653-03D5V and PX653-10D5V pressure transducers. The air is then passed through a high efficiency particulate air (HEPA) filter. The air then mixes with test aerosol of nano-sized particulates of NaCl dissolved in deionized water generated via a TOPAS ATM241 aerosol generator. A dilution loop regulates the concentration of the test aerosol. The test aerosol then passes through the mounted respirator at a filter face velocity of 16.5 cm/s for high-flow and 5.5 cm/s for low-flow which match flow rates specified in CFR guidelines [28]. The entire area of the respirator was tested in the LSFS. Probes are used to measure the pressure on either side of the respirator. A TSI Scanning Mobility Particle Sizer Spectrometer (SMPS) 3938 measures the size and quantity of particles on either side of the respirator by sampling the air through the isokinetic probes. The scanning system can collect data for particle sizes well below 75 nm, the particle size referred to in NIOSH guidelines [29], and above the 300 nm FDA recommend mass median particle diameter. Documented size distributions of SARS-CoV-2 aerosol range from of 250 nm to greater than 25 μm [30]. The bill shaped respirator, Model 2, was tested on this machine. This system has since been validated in other studies against a COTS instrument designed to meet the NIOSH N95 testing criteria [31, 32].

Both systems use similar TSI instruments and components, so it is reasonable to compare results between machines. The FPT can separate the particle selection (Electrostatic Classifier/Differential Mobility Analyzer) and the particulate counter (Condensation Particle Counter). The LSFS combines these to run a full particle size distribution scan with concentration. For both machines, efficiency reduction is defined as the efficiency of the 25 kGy samples minus the efficiency of the control samples. Efficiency is calculated at each particle size by taking the concentration present downstream of the respirator and dividing it by the concentration upstream of the sample. This is the performance metric shown in our results.

### Electrostatic potential measurements

Many newer N95 respirators use an electret material to efficiently capture particles out of the air using electrostatic forces [33]. Due to the ability of gamma radiation to modify electret material charge, the purpose of this test was to have a quantifiable method of measuring this factor [19, 34].

For electrostatic measurements, 47 mm outer diameter samples were taken from each respirator. These samples were separated into 3 layers for Model 1 and 4 layers for Model 2. The layers can be further separated, but not without damaging the filter media, which we believe would alter the electrostatic measurement. Surface potential measurements were made using two electrostatic voltmeters which were grounded to the surface of a metal optical table. The Trek 344 electrostatic voltmeter uses a non-contact probe and has a maximum potential reading of 2000 V. The Trek 821HH uses a contact probe and has a maximum potential measurement of 2430 V. The two probes average over differing measurement areas but utilize a similar potential following method [35]. The non-contact method is more common in the literature, but frequently reached the maximum potential reading on the electret materials. The contact voltmeter probe measured a consistently lower surface potential on respirator materials and sometimes punctured the thinner (non-filter) layers, but the two methods measured similar relative changes in surface charge. Repeatability and accuracy were tested against a 1” aluminum disc connected to a DC power supply and by measuring the grounded table surface (both voltmeters measured the correct magnitude on these references within ±1 V).

Each respirator sample was equilibrated at room environment (controlled temperature 20–21°C and with relative humidity in the range of 45–51%) for 30 minutes on a grounded surface prior to testing. The samples were mounted between three electrically isolated alligator clips on the edges of the samples above the surface of a grounded metal optical table. A Fluke 289 multimeter was used to verify that the insulated surface was not electrically connected to the field-meter ground. The non-contact voltmeter was placed ~2 mm away from the surface and the contact voltmeter was gently touched to the surface of the fabric sample. Measurements were made in four locations away from the attachment points on both sides and averaged.

To identify the layer of each respirator likely to contain the electret filter media, a Keyence VHX6000 microscope was used to image each layer of the control respirators. Electret filter media are made of meltblown polypropylene, which looks like fibrous material under the microscope.

## Results

We observe that filtration performance was degraded after 25 kGy in both models of N95 respirators. Electrostatic potential was measured on each respirator layer. Statistically significant changes were observed in layers with electret filter media. Results for the aerosol filtration performance testing and electrostatic potential measurements are presented below.

### Aerosol filtration performance testing

**Fig 6:**
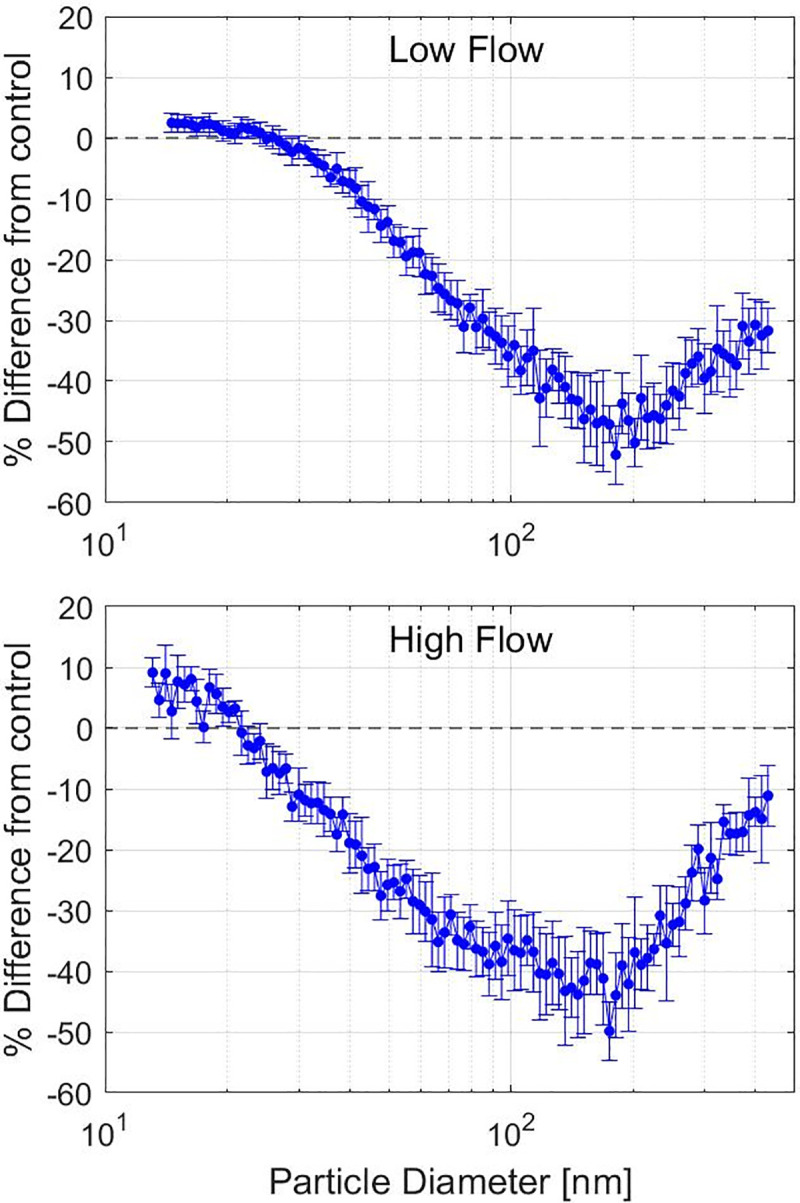
Efficiency reduction of irradiated Model 2 respirator. Efficiency reduction of 25 kGy(tissue) Model 2 respirator when compared to control respirator on the LSFS. Note that the small particle sizes in the range of 10–20 nm are heavily influenced by many factors (such as temperature) and the slight increase in efficiency needs further investigation.

Efficiency reduction for the irradiated Model 1 respirator are reported in **Table 2** for the challenge particle size of 75 nm. The Model 2 respirator efficiency reduction data for the LSFS are shown in **Fig 6**. These results show that gamma irradiation substantially reduces the efficiency of respirators to filter particle sizes in the range of 75–300 nm.

**Table 2:** Efficiency reduction of Model 1 respirator.

Sample	Efficiency Reduction [%]
1	-42 (5)
2	-45 (6)

Model 1 respirator efficiency reduction measurements with particulate size 75nm for the FPT. Standard deviation in parentheses.

### Electrostatic potential measurements

The measured surface electrostatic potential for each respirator layer is shown in **Table 3** for the contactless Trek 344 measurements. 2000 V is the maximum measurable potential, thus limiting our ability to measure the percent change. The data in **Table 4** shows the contact results from the Trek 821HH. Both methods had significant variability in the measurements (when not hitting the maximum reading) as shown by the standard deviations of the measurement in both tables. Significant variability in field measurements is consistent with other literature measurements of electret materials [36]. While the absolute values are uncertain, we are confident that the two methods qualitatively detected changes in the charge state of the respirator layers before and after exposure to gamma radiation. Additional information and charts about the electrostatic potential measurements can be found in **S2 File**.

**Table 3:** Trek 344 electrostatic potential measurement of various layers within respirators.

Models	Layer	Average 0kGy(tissue) (V)	Average 25kGy(tissue) (V)	Percent Change
1	1 (Outer)	-2000 (0)	-2000 (0)	N/A
	2	**2000 (0)**	**-2000 (0)**	**N/A**
	3 (Inner)	0 (0)	0 (0)	-
2	1 (Outer)	-1560 (590)	-630 (450)	60%
	2	**650 (780)**	**-2000 (0)**	**N/A**
	3	-1950 (100)	1040 (760)	153%
	4 (Inner)	0 (3)	-3 (2)	-

Standard deviation given in parenthesis.

**Table 4:** Trek 821HH electrostatic potential measurement of various layers within respirators.

Models	Layer	Average 0 kGy(tissue) (V)	Average 25 kGy(tissue) (V)	Percent Change
1	1 (Outer)	-2040 (520)	-1374 (150)	33%
	**2**	**1590 (270)**	**-1980 (340)**	**225%**
	3 (Inner)	1 (1)	0 (1)	-
2	1 (Outer)	-1100 (160)	-320 (90)	71%
	**2**	**650 (190)**	**-1470 (160)**	**328%**
	3	-930 (80)	820 (290)	171%
	4 (Inner)	5 (2)	7 (4)	-

Standard deviation given in parenthesis.

Electrets are meltblown polypropylene that creates fibrous layers. Both respirator models have a pair of fine fiber electret layers in their core that are the primary means of filtration (shown in bold in **Tables** **3** and **4**). Microscopy found that layer 3 of Model 2 was not a fibrous layer. Rather, it was a solid layer with 300 micrometer sized holes (**S8 Fig** in **S1 File**). Electrostatic potential testing prior to gamma irradiation showed that the filtration layer had significant electrostatic potential and it appears that the positively charged filtration layers in both respirators changed their sign after exposure to gamma radiation.

Although our experiment design did not include macroscopic visual or tactile measurements of the respirator, we did not notice qualitative changes. While preparing individual layers for analysis, we noted that the filtration layer encapsulated within each respirator became more fragile and friable after gamma radiation exposure. This observation was made in other gamma studies of N95 respirators as well [26].

## Discussion

This study utilized aerosol filtration performance and electrostatic measurements to detect changes in efficiency of N95 respirators after applying 25 kGy(tissue) of dose. Other tests of respirator efficiency, such as Ambient Aerosol Condensation Nuclei Counter Quantitative Fit Test, are common and accessible methods to determine mask appropriateness for individuals. Quantitative fit tests, however, are intended for respirator fitting of pristine materials and are limited in how much information they can give on the degradation of a mask. They can only determine whether a respirator fits and is appropriate for a user; they cannot determine to what degree a mask filters nor why it is damaged. The tests used in this study were chosen to understand the degree of degradation and correlate those with electrostatic changes in the filter material.

Our results demonstrate that N95 respirators exposed to gamma radiation exhibit a reduction in filtering efficiency. The filtering efficiency was compromised for both respirators, to varying degrees. These results are consistent with previous research that found a reduction in filtration efficiency in the particle size range 100–300 nm [17, 23, 24]. Note that this particle size range is relevant to disease transmission [25].

The reduction in filtration efficiency was accompanied by changes in electrostatic charges of the filtration layer within the respirators. However, it is worth noting that the small sample size and large variability of electret material field measurements [36] made it challenging to obtain high certainty quantitative measurements. Nevertheless, our findings are consistent with other studies that have observed degradation in filtration performance associated with electret material degradation for other sterilization methods, such as isopropanol, ethanol, and chlorine based methods [7, 8]. In one study, filtration efficiency reduction (via TRIGA reactor exposure) was correlated with removal of charge in the respirator [26]. Our results replicated this reduction in filtration efficiency using cobalt-60 gamma radiation. We expand upon the previous study’s measurements by providing electrostatic potential measurements on each individual layer of the respirator. Whereas the previous study measured a net removal of charge on the entire respirator, our measurement of the isolated electret layer shows a substantial change in potential and a switch in polarity. Both models tested in this study had electret layers and we were unable to compare these models to respirators that did not rely on electret material for filtration. Comparing our results to respirators without electret filtering materials would further support that the modification of charge on the electret layer is responsible for the loss of filtering efficiency.

Further, the small sample size of two models limits the applicability of this research to all N95 respirators. Because N95 respirators depend on different filtering layers and technologies within the layers, it is likely that each make and model of respirator would need to be analyzed for damage after sterilization doses of irradiation and any other form of sterilization that main impact the electret layer.

As in other studies, we did not observe any signs of visible damage in the respirators after irradiation [23]. Because of this, detecting change in the electret layer potential can serve as an indicator of filter efficiency degradation. Our results demonstrate methods to detect changes in the electret layer potential that would be one indicator of substantial change in filtration performance. Notably, other forms of PPE do not contain electret layers and may be better suited for gamma sterilization in shortage conditions.

## Conclusions

The collection of tests performed on the respirators revealed that gamma radiation can modify the electrostatic charge on the filter and this change coincided with a decrease in aerosol filtering efficiency around the 75–300 nm particle size.

Although gamma irradiation is a well-known technique for medical sterilization, and has many potential advantages (facility availability, penetration, handling, and safety), it is beneficial to test for effects in performance and integrity after gamma sterilization. The respirators were degraded in performance, but had no significant visual, or tactile indicators of the degradation. The electrostatic potential change in the electret layer can be measured with contact and non-contact probe methods and can be an initial indicator of respirator performance degradation.

## Supporting information

S1 File Imaging of control respirators. (DOCX)

S2 File Electrostatic potential data. (XLSX)

S3 File Filter penetration testbed data. (XLSX)

S4 File Large-scale filtration system data. (XLSX)
