# Supplementary material for: Gamma radiation sterilization of N95 respirators leads to decreased respirator performance
Source: PLoS One. 2021 Apr 8;16(4):e0248859. doi: 10.1371/journal.pone.0248859 (PMC8031388; doi:10.1371/journal.pone.0248859)
Supplement: S1 File — (DOCX) [file pone.0248859.s001.docx]

**S1 File. Imaging of Control Respirators.**

Microscopic imaging of individual layers of control respirators was completed using a Keyence VHX6000 microscope.


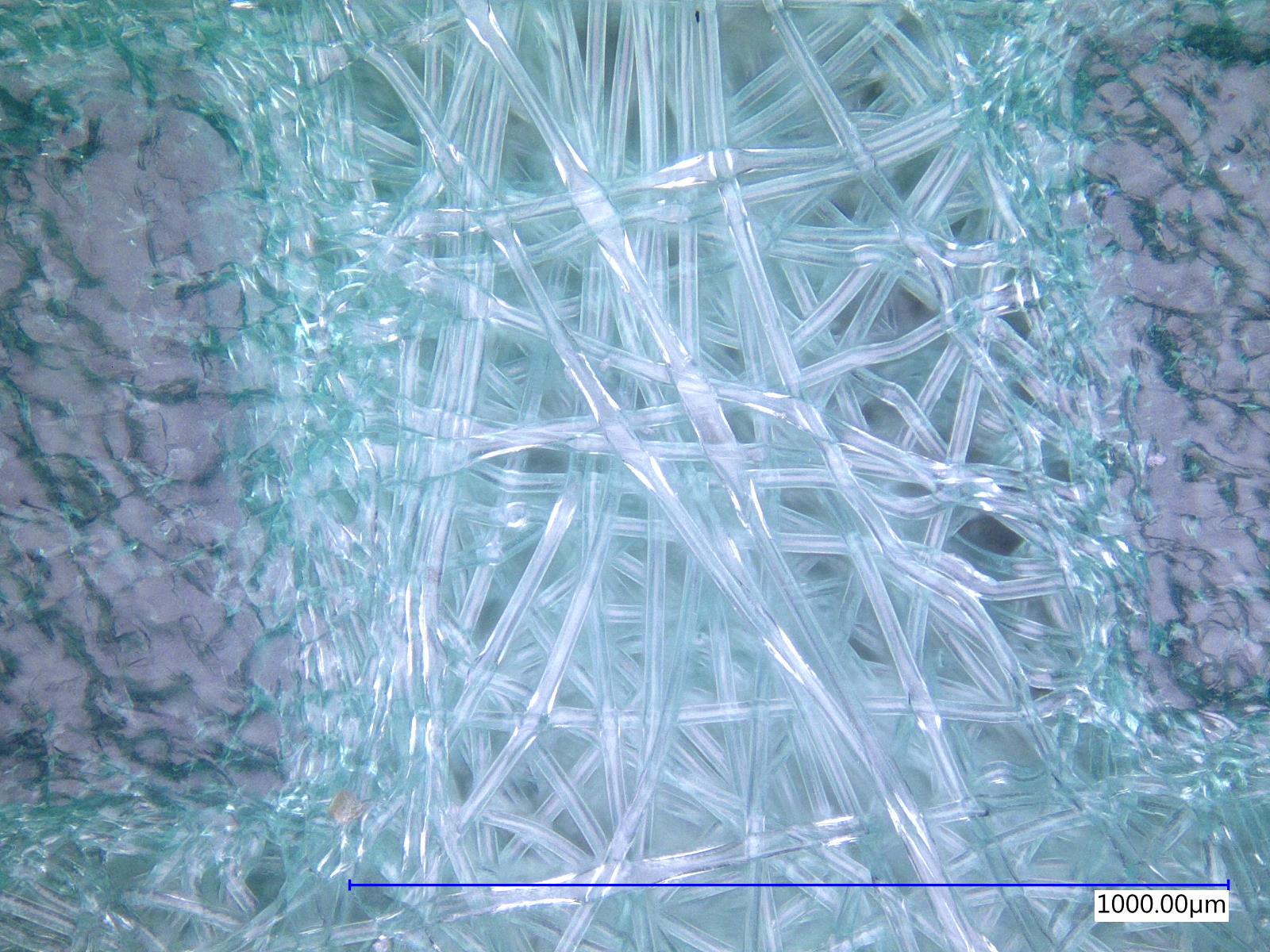


S1 Fig. Model 1 N95 Layer 1 250x 0 kGy(tissue).


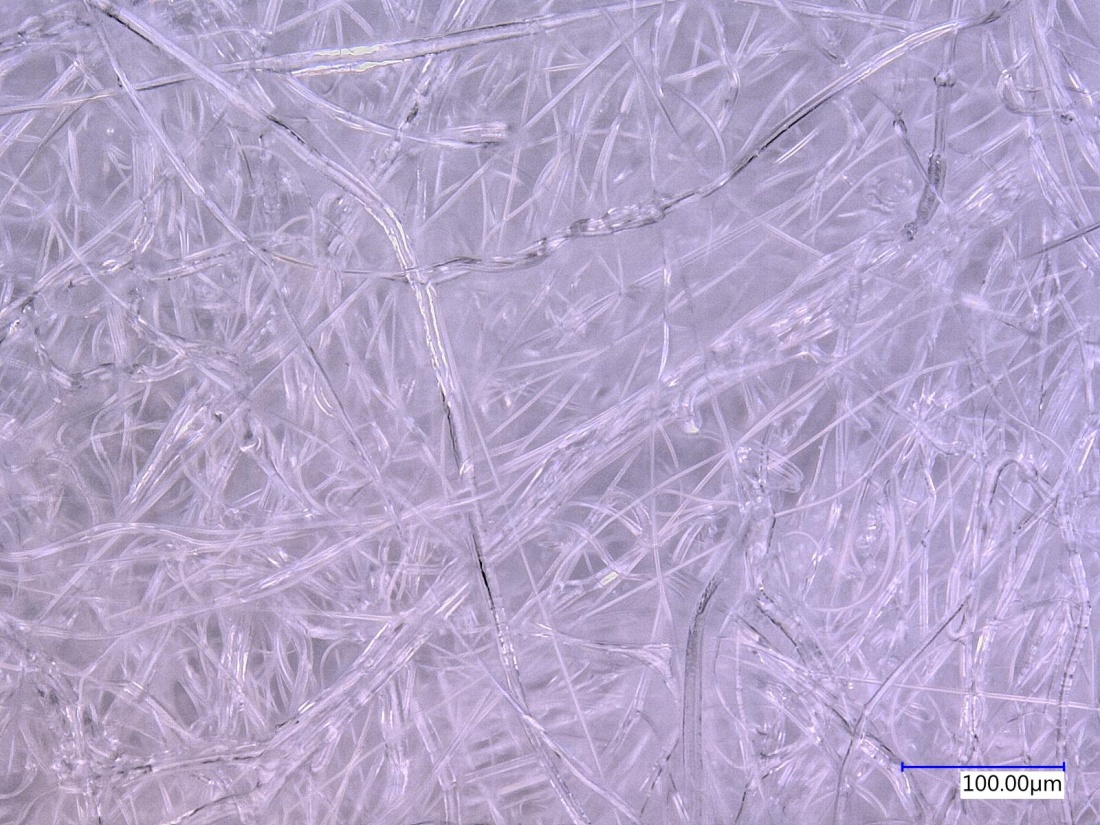


S2 Fig. Model 1 N95 Layer 2 500x 0 kGy(tissue).


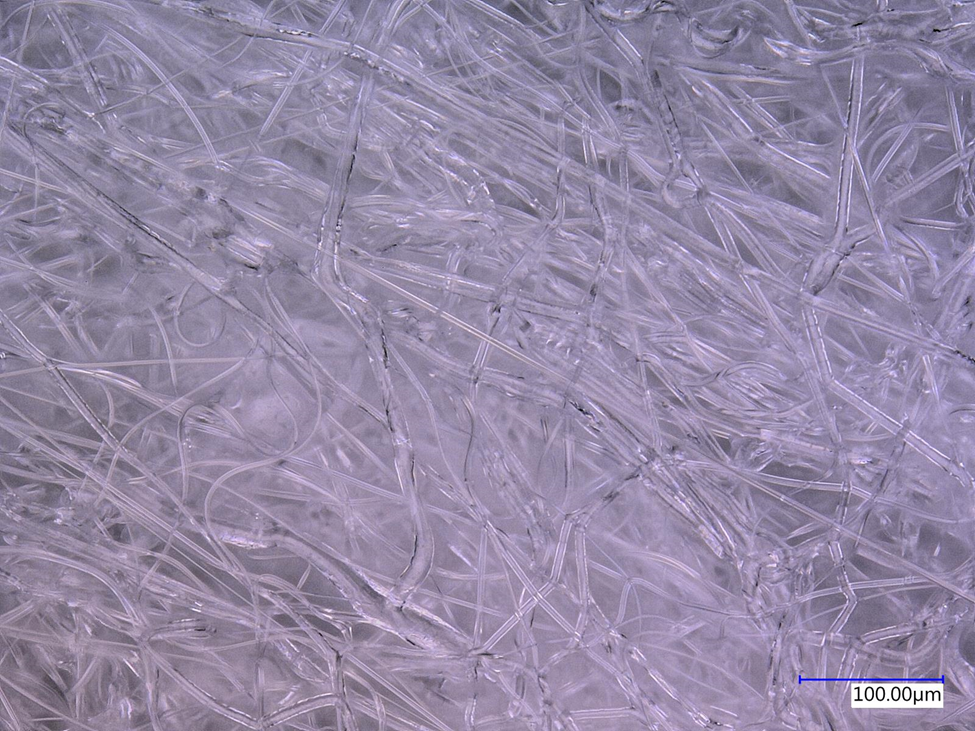


S3 Fig. Model 1 N95 Layer 3 500x 0 kGy(tissue).


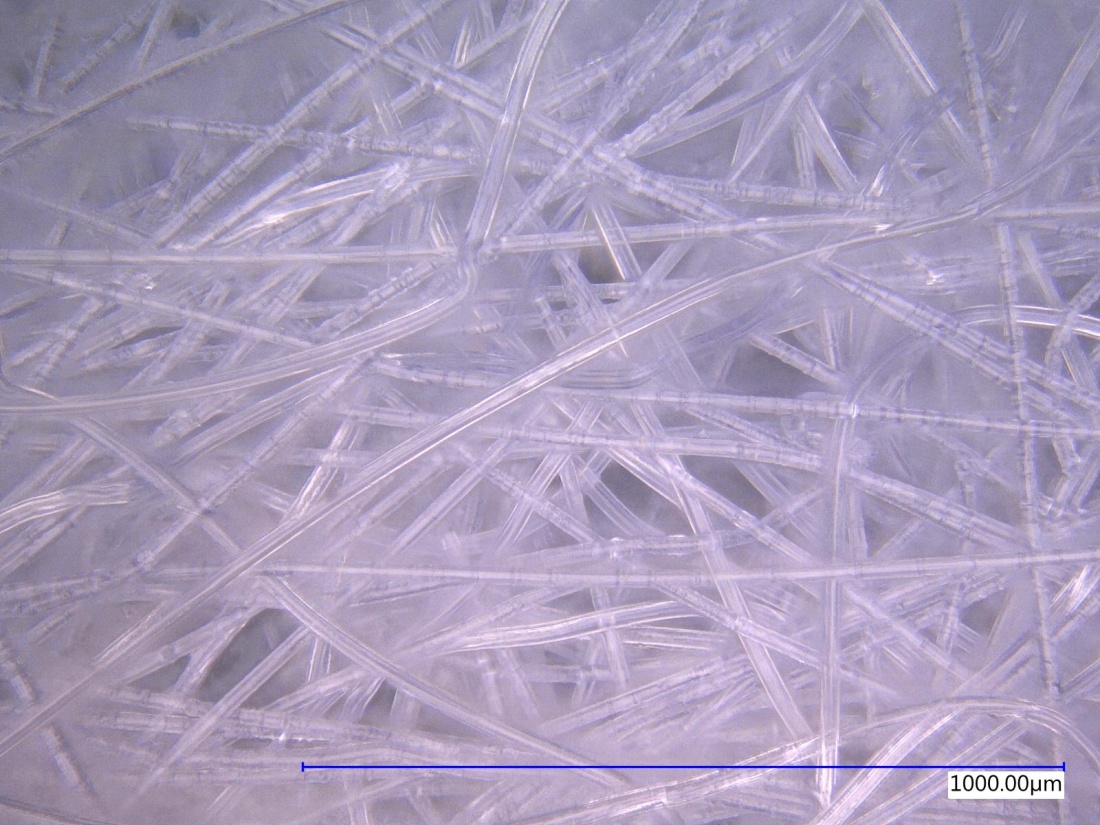


S4 Fig. Model 1 N95 Layer 4 250x 0 kGy(tissue).


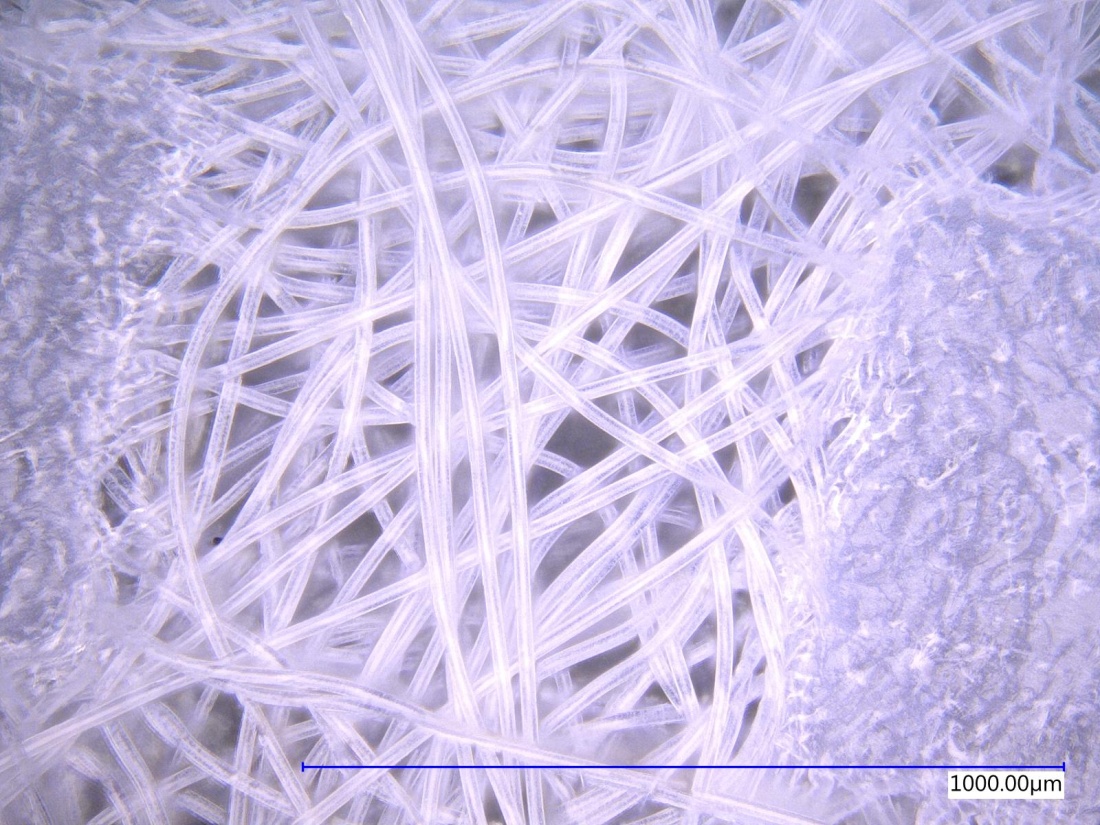


S5 Fig. Model 2 N95 Layer 1 250x 0 kGy(tissue).


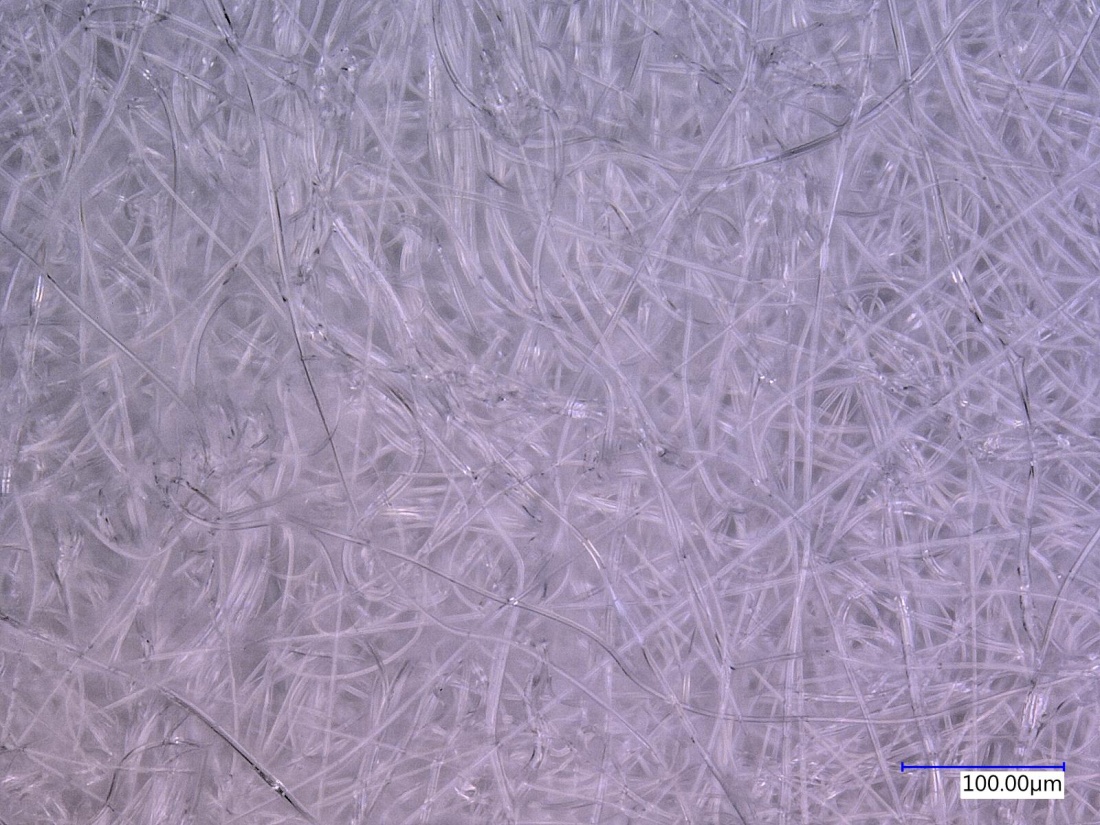


S6 Fig. Model 2 N95 Layer 2a 500x 0 kGy(tissue). Note: Layer 2 separated into two sub-layers, although the fibrous nature of this layer led to damage during the separation. We took pictures of individual sublayers. Electrostatic potential measurements were conducted on layer two as a whole to ensure the splitting did not affect measurements.


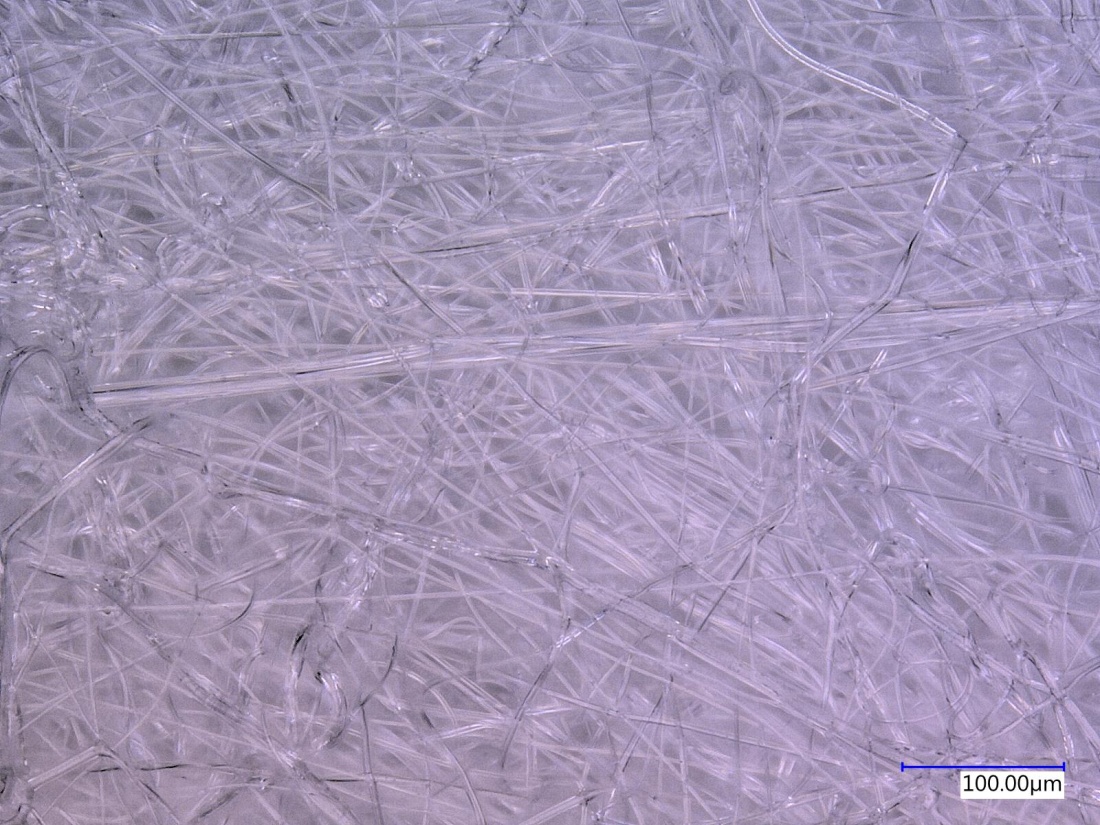


S7 Fig. Model 2 N95 Layer 2b 500x 0 kGy(tissue). Note: Layer 2 separated into two sub-layers, although the fibrous nature of this layer led to damage during the separation. We took pictures of individual sublayers. Electrostatic potential measurements were conducted on layer two as a whole to ensure the splitting did not affect measurements.


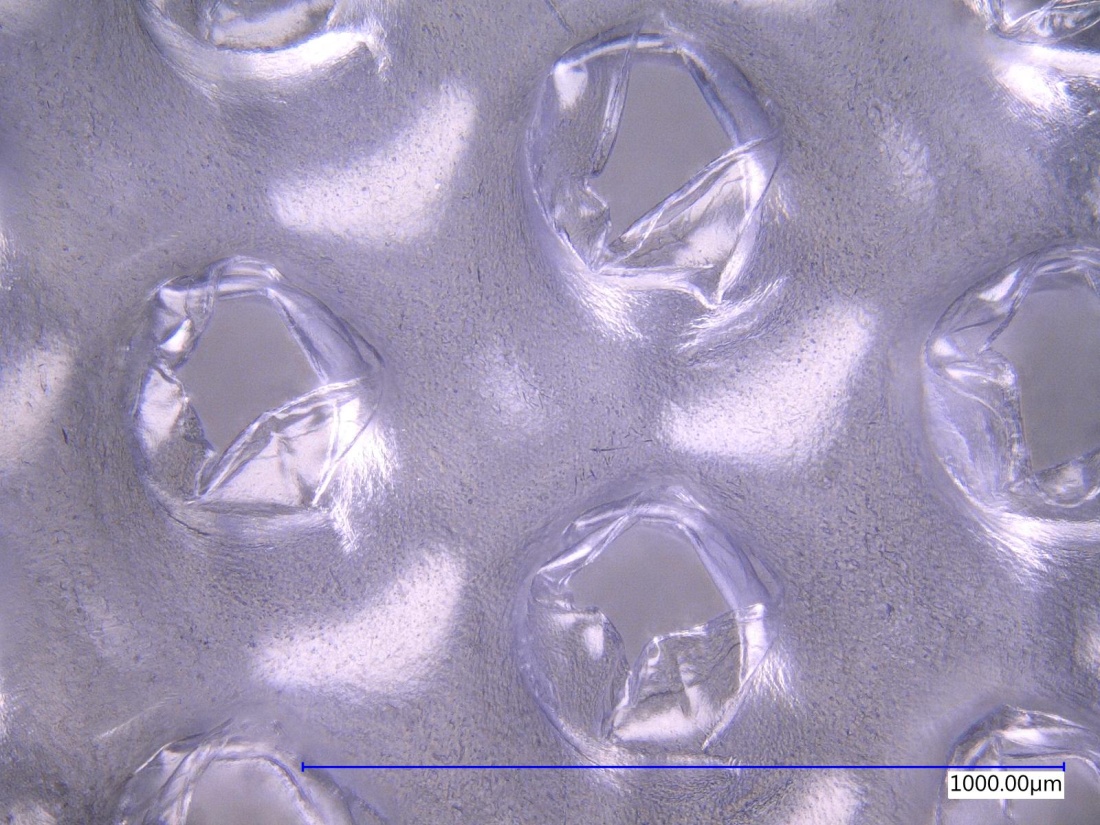


S8 Fig. Model 2 N95 Layer 3 250x 0 kGy(tissue).


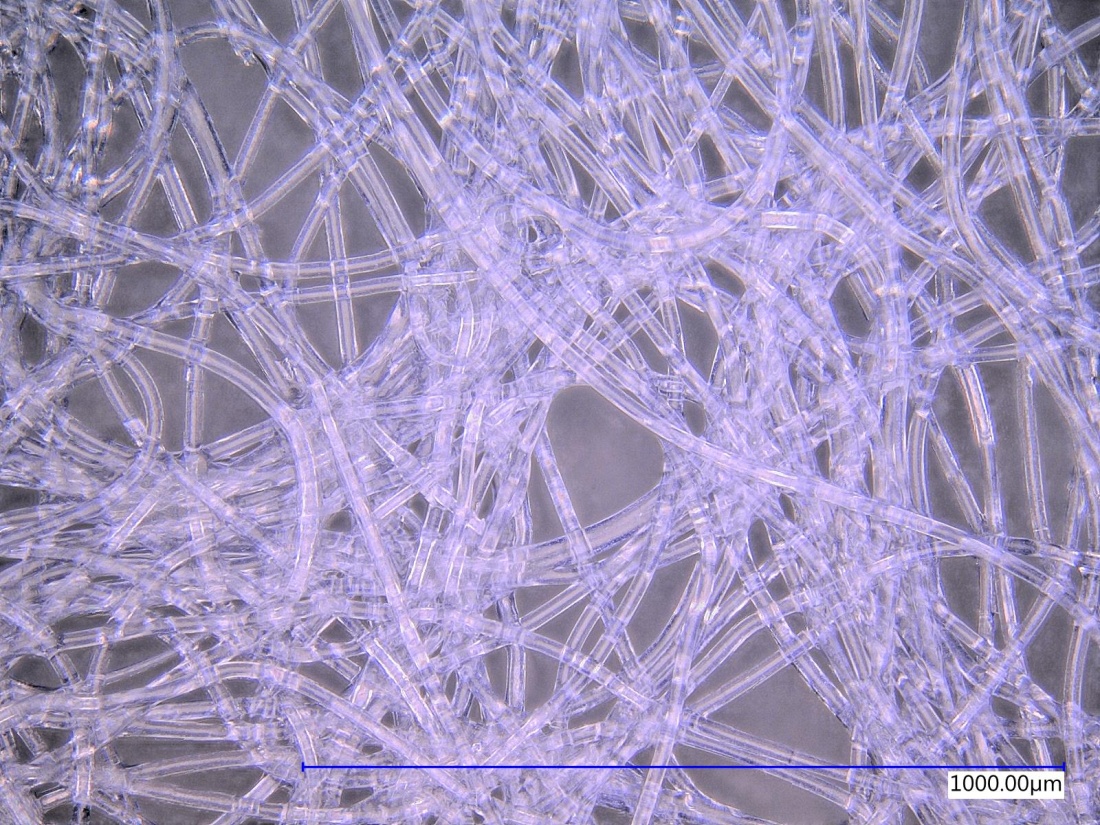


S9 Fig. Model 2 N95 Layer 4 250x 0 kGy(tissue).
